# Sweating as a Preventive Care and Treatment Strategy in Traditional Persian Medicine

**DOI:** 10.31661/gmj.v9i0.2003

**Published:** 2020-12-25

**Authors:** Mahboubeh Mahlouji, Mahdi Alizadeh Vaghasloo, Majid Dadmehr, Hossein Rezaeizadeh, Esmaeil Nazem, Haleh Tajadini

**Affiliations:** ^1^Neuroscience Research Center, Institute of Neuropharmacology, Kerman University of Medical Sciences, Kerman, Iran; ^2^Department of Traditional Medicine, School of Persian Medicine, Kerman University of Medical Sciences, Kerman, Iran; ^3^School of Persian Medicine, Tehran University of Medical Science, Tehran, Iran; ^4^School of Persian Medicine, Iran University of Medical Sciences, Tehran, Iran

**Keywords:** Traditional Persian Medicine, Sweating, Diaphoretic Herbs, Herbal Medicine

## Abstract

Proper and regular sweating plays a significant thermoregulatory role. It is a common perception that, sweating has other important homeostatic functions such as clearance of excessive micronutrients, waste products of metabolic processes, and toxins from the body, which helps to maintain human good health. In addition, sweating, thermotherapy, and sauna are commonly used to treat various diseases such as cardiovascular, respiratory and joint diseases. In traditional Persian medicine (PM) textbooks, sweating is considered a preventive care and treatment strategy as well. In this study, we aim to explain the beneficial effects of sweating in human health and its role in the management of various diseases, as well as introducing the therapeutic applications of some diaphoretic plants from the viewpoint of PM. We reviewed the most famous PM textbooks such as *Kamil al-Sinaa al-Tibbiya*, *Al-Qānūn fī al-Tibb*, *Zakhireye Kharazmshahi*, *Kholasat al-Hikmat*, *Exir-e-Azam*, and *Hifzos-sihhat-e Naseri*. Also, current evidence was searched in PubMed, Web of Science, Scopus, and other relevant databases related to the topic. The results of this study revealed that PM scientists believed proper sweating removes waste products and maintains the body’s health, thus, any disturbances in the excretion of these waste products can cause diseases. They recommended the induction of sweating through hot and dry baths, sun bath, sand bath and also the use of diaphoretic herbs for the management of various diseases. Therefore, further researches are recommended to evaluate the effectiveness of these diaphoretic plants.

## Introduction

Sweating has an important role in regulating human body temperature and this physiological effect is observed in periods of intensive physical activity or exposure to warm environments [1]. Furthermore, regular sweating is responsible for other types of important homeostatic functions, including the clearance of excessive micronutrients, waste products of metabolic processes, and toxins from the body [2]. Despite the important role of normal sweating in maintaining human health, inability to sweat normally or excessive abnormal sweating can cause significant health concerns. Hyporhidrosis or pathologic anhidrosis which are inadequate or inability to sweat respectively, may cause symptoms of dry skin, heat exhaustion, heatstroke, and even death. Also, hyperhidrosis, a condition that results in excessive sweating, is associated with some systemic diseases or anxiety that has negative effects on patients’ quality of life [3]. Numerous studies have shown that proper sweating, in addition to preventing some diseases, can reduce their symptoms and improves patients’ quality of life as well. Several studies demonstrated clinical effects of induced sweating methods such as thermotherapy, waon therapy and sauna in common diseases such as cardiovascular [4,5], respiratory [6,7], and joint diseases [8], and chronic pain [9], as well as in prevention and risk reduction of dementia and Alzheimer’s disease [10]. Waon therapy is a form of thermal treatment in a dry sauna, which the entire body is warmed. This modality improves hemodynamics, ventricular arrhythmias and vascular function in patients with congestive heart failure (CHF) [5]. Reviewing historical medical manuscripts indicates that traditional Persian medicine (PM) scientists have described several methods for the treatment of diseases. Sweating is one of them which has an important role in both prevention and treatment of diseases. PM physicians were well aware of the health benefits of sweating and believed that sweating removes waste products, maintains the body health, and balances body temperature. Based on the principles of PM, any disturbances in the excretion of metabolic and dietary waste products can cause disease; therefore, the use of several sweating methods and even diaphoretic herbs have been considered in maintaining human health and as one of the therapeutic method since many centuries ago [11]. To the best of our knowledge, this topic has not been fully studied; therefore, we aim to explain the beneficial effects of sweating in human health and its role in the treatment of diseases, as well as introducing therapeutic applications of some diaphoretic plants from the viewpoint of PM.

## Search Strategies

In this study, we reviewed the chapters related to sweating and diaphoretic plants in several PM textbooks, including *Kamil al- Sinaa al- Tibbiya* (10th Century), *Al- Qānūn fī al- Tibb* (11th Century), *Zakhireye Kharazmshahi* (12th Century), *Kholasat al- Hikmat* (18th Century), *Exir -e-Azam* (19th Century), and *Hifzos - sihhat -e Naseri* (19th Century). The search was performed with the keywords “ *Ta’riq*”, “ *Taarroq*”, “ *Mo’arriq*”, “ *Araq*” and “ *Hammam*”. Diseases of the human body, which sweating was suggested in their treatment, were categorized. Also, the diaphoretic herbal medicines used in the treatment of diseases were gathered. We collected and classified the items related to the subject. Furthermore, a search in PubMed, Web of Science, Scopus, and other relevant databases from June 2002 up to June 2020 was accomplished to review recent advances in this field. The keywords of the search were “sweating”, “sweat”, “bath”, “diaphoretic herbs”, “perspiration”, “thermotherapy”, “sauna” and other related terms.

## Results

From the perspective of PM, sweat contains waste products that are produced during the digestive process in the human body. In general, foodstuff goes through four stages of digestion to be converted to a matter fit for assimilation. These stages are known as gastric, hepatic, vascular, and tissue digestion. In each of them, different waste products are also produced, which must subsequently be excreted from the body. Excretion of the waste products in the gastric and hepatic digestion occurs via feces and urine respectively. The metabolic waste products of vascular and tissue digestion stages are excreted via sweat [12,13]. Sweat is believed to be the excess materials produced in the third and fourth stages of digestion which are excreted via skin pores namely “ *Masam*” [14]. These pores are a lot of small holes throughout the skin that sweat comes out of these narrow pores and reaches the surface of the skin [11,12,15].

###  Application of Sweating in the Preservation of Health

Based on the viewpoint of PM, preservation of health “ *Hifzos-sihhah*” has an important role and precedes treatment. PM scientists believed that six essential principles called “ *Sittat od- daruriyyah*” must be considered to preserve health and prevent diseases as well. These principles cover various aspects of human life and include climate, food and drink, sleep and wakefulness, motion and stillness, psychological and mental reactions and evacuation and retention. The equilibrium between these six principles is essential to have a healthy lifestyle. Among them, regular evacuation and cleansing of waste products from the body, which is done via various routes, including stool, urine, and sweat is of special importance [11,13,16]. A healthy person is expected to have regular and adequate sweating to stay healthy. Therefore, any disturbance in the excretion of waste products, can cause disease and disrupt health conditions [11,17]. Therefore, in the preventive approach and healthy lifestyle management proper sweating is a simple, safe, and accessible method that is recommended in PM sources. In a healthy person, sweating occurs during moderate exercise, daily physical activity, exposure to hot season or climate, and regular use of baths to maintain health and prevent diseases [11].

###  Application of Sweating in the Treatment of Diseases

According to PM teachings, the accumulation of waste products in different parts of the body can lead to disease. Therefore, the excretion of these substances from the body’s natural excretory pathways is a therapeutic strategy. One of the recommended methods of treatment is the induction of sweating through the use of hot and dry baths, sun bath, sand bath (to be concealed), and also the use of diaphoretic herbs [11,12,18]. PM physicians have considered the use of sweating as one of the treatment options for various diseases. Most of these diseases are caused by the accumulation of excessive moisture in the body. They include diseases of various organs such as the brain, gastrointestinal tract, heart, respiratory tract, kidneys, musculoskeletal and skin, also poisoning, and bites [18]. Some of them are shown in Table-1.

###  Diaphoretic Herbal Medicines

Diaphoretic are those herbs that cause sweat to come out of the skin pores. These plants have special properties that cause skin pores to open and pass diluted waste products under the skin through these pores [13,19]. These plants are used locally (embrocation) and orally (decoction) and are consumed in singular or compound drugs [19]. Some of the most important diaphoretic plants are summarized in Table-2.

## Discussion

According to the PM textbooks, some waste products are produced throughout the four-step process of digestion in the human body. It is clear that failure to excrete these substances and their accumulation in various organs causes dysfunction and diseases of those organs, therefore, they must be expelled from the body. These wastes are excreted from the body in various ways, including feces, urine, and sweat. Any change in regular and adequate sweating in the human body can contribute to the development of the disease in the future. Sweating excreted the waste products in metabolic processes and toxins from the human body, and promotes the health. Sweat is excreted through the very small pores on the surface of the skin [11,13]. These pores seem to correspond with pilosebaceous units of sweat excretion from eccrine and apocrine glands, which can import and export substances and drugs and are effective in heat lowering. In other words, they play an important thermoregulatory role [20,21]. PM scientists believed that abnormalities in the sweating process can lead to diseases such as humid and phlegmatic brain diseases, phlegmatic and sanguineous strokes, paralysis, phlegmatic headache, diabetes, and phlegmatic joint pain, so, administration of proper sweating is considered one of the most effective treatments in these diseases [11,12,18]. Current studies also demonstrate that normal sweating removes waste products and toxins from the body and is associated with maintaining good health and preventing diseases [22]. Many studies have been done on the use of sauna, waon therapy, and thermal therapy as a choice treatment for some diseases. These methods have been considered safe and effective [23-26]. A reduced risk of dementia and Alzheimer’s disease in middle-aged men [10], as well as a reduced risk of stroke in middle-aged and older men and women, who frequently use sauna have been reported [27]. Numerous studies have suggested the effect of sauna in the treatment of various cardiovascular diseases. This effect can be associated with the reduction of oxidative stress and subsequent atherosclerosis, which is achieved through sauna therapy [28]. In patients with coronary risk factors, sauna therapy is related to the improvement of vascular endothelial cell function [23,29]. The sauna bathing has shown beneficial effects on some cardiovascular diseases including improved ventricular arrhythmias, ameliorating of clinical manifestations and heart function, decreased heart size, and increased left ventricular ejection fraction (EF), significant improvement in blood pressure in hypertensive people, improved chronic heart failure (CHF) symptoms and prognosis, and decreases in sudden cardiac death [30,31]. There are ameliorating effects in myocardial perfusion after repeated sauna treatment in patients with chronic coronary-related ischemia [29]. The use of thermal therapy on respiratory diseases shows an improvement of the respiratory function in patients with allergic rhinitis, improving pulmonary hypertension, and also airway obstruction in patients with COPD [6,32,33]. Long-term prospective studies display that a sauna bath can be effective in reducing the risk of pneumonia [7,34]. Thermal therapy is effective in improvement in pain severity and fibromyalgia symptoms. Moreover, sauna therapy has improved chronic fatigue syndrome and chronic pain, as well as can effectively reduce the symptoms of rheumatoid arthritis and ankylosing spondylitis [8,9,35,36]. Thermal therapy and sauna affect lipid profile and quality of life in patients with type 2 diabetes mellitus and obesity [37-39]. Regular sauna bathing has been considered effective in the management of tension headaches [40]. Sauna-based detoxification therapy reduced the chronic symptoms related to the chemical exposures of methamphetamine and also improved the quality of life [41].

## Conclusion

 In both traditional PM and conventional medicine, proper and regular sweating is considered as a preventive care and treatment strategy. According to the PM textbooks, several methods have been proposed for the treatment of diseases and the use of sweating is one of them. By inducing sweating, some diseases of the body organs are either completely cured or sweating has significant effects on their improvement. PM physicians believed that sweating, hot and dry baths, and diaphoretic herbs as a simple, safe, and affordable method can be used to prevent and treat diseases. Therefore, further researches are recommended to evaluate the effectiveness of these diaphoretic plants.

## Acknowledgment

 This paper is part of Ph.D. thesis of Dr. Mahlouji (Thesis number: 95-10-27-91).

## Conflict of Interest

 The authors declare that they have no conflict of interest.

**Table 1:** Some Diseases which are Treated with Sweating in the PM Sources.

** Respiratory system [11,18] **	Cough (phlegmatic, cold, humid) Catarrh / *nazlah* / Fine and short sound Epidemic diseases due to putrifactive air
** Caediovascular system [11] **	Faint /ghashy/(related to *Imtila*)
** Urogenital system [11,18] **	Amenorrhea due to obesity Renal emphysema Dysuria
** Fever and infectious diseases [11,12,18] **	Hot fever Hyperpyrexia Septic fever Phlegmatic fever Quartan fever Phlegmatic atrabiliary fever Phlegmatic quartan fever Types of diurnal fever /homma al-yawm/ Synochus (paratyphoid) fever / *sunukhas*/
** Skin [11,12,15,18] **	Vitiligo / *baras*/ Vitiligo alba / *baras ol-abyad*/ Pediculosis Phlegmatic urticaria Miliaria rubra Pruritus Creeping ulcers Scabies Leprosy
** Gastrointestinal track [11,12,18] **	Diabetes / *dhiyabitos*/ Severe fatness / *siman mofrit*/ Dropsy, anasarca, ascites / *Istisqa*/ Hiccough / *fowaq*/ Diarrhea Cold colic /qulanj-e barid/ Splenic tumefactions Jaundice Intestinal abrasion /sahj/
** Central and periferal nervous system [12,15,18] **	Phlegmatic brain diseases Brain diseases due to moisture Hemicranial headache / *shaqiqah*/ Dizziness / *sadar*/ Vertigo / *dowar*/ Phlegmatic headache Unilateral facial paralysis / *laqwah*/ Numbness / *khadar*/ Paralysis / *falij*/ Phlegmatic apoplexy Senile heterotropia Tremor/ *ra’shah*/
** Musculo-skeletal system [11,12,18] **	Phlegmatic arthralgia Gout / *niqris*/ Spasm / *tashannoj*/ (cold, humid)
** Poisoning and bites [11,12,18] **	Poisoning Snake bites Rabid dog bites Scorpion sting Spider bites

**Table 2:** Diaphoretic Herbal Medicines Suggested in the PM Sources [11,12,19].

**Scientfic name**	**Family**	**Common name**	**Persian name**	**Used part**
*Agrimonia eupatoria L.*	Rosaceae	Agrimony	Ghafas	All parts
*Allium sativum L.*	Amaryllidaceae	Garlic	Sowm	Root/bulb
*Urtica dioica L.*	Urticaceae	Common Nettle	Anjoreh	Seeds/ leaves
*Apuim graveolens L.*	Apiaceae	Celery	Karafs	Seeds/root/aerial parts
*Artemisia absinthium L.*	Asteraceae	Common wormwood	Afsantin	Leaves/flowering tops
*Carum copticum L.*	Apiaceae	Ajwain	Nankhah	Seeds
*Cinnamomum citriodorum Th.*	Lauraceae	-	Sazaj	Leaves
*Cucumis melo L.*	Cucurbitaceae	Melon	Bettikh	Fruits/seeds
*Cuscuta monogyna Vahl.*	Cucurbitaceae	-	Koshous	Seeds
*Ficus carica L.*	Moraceae	Common fig	Tin	Fruits
*Linum usitatissimum L.*	Linaceae	Common flax	Kattan	Seeds
*Matricaria chamomilla L.*	Asteraceae	German chamomile	Babounaj	Flowers
*Mentha pulegium L.*	Labiatae	Pennyroyal	Foudanaj	Leaves
*Ocimum basilicum L.*	Lamiaceae	Sweet basil	Reihan	Leaves
*Tanacetum parthenium*	Asteraceae	Feverfew	Oqhowan	Flowers
*Pimpinella anisum L.*	Apiaceae	Anise	Anisoon	Seeds
*Trifolium alexandrium L.*	Papilionaceae	Egyptian clover	Handaghoughi	Roots, seeds, leaves
*Myrtus communis*	Myrtaceae	Mourd	Myrtle	Leaves /fruits
